# Complete Genome Sequence of the *Streptococcus suis* Temperate Bacteriophage ϕNJ2

**DOI:** 10.1128/genomeA.00008-12

**Published:** 2013-01-24

**Authors:** Fang Tang, Alex Bossers, Frank Harders, Chengping Lu, Hilde Smith

**Affiliations:** a Key Lab Animal Bacteriology, Ministry of Agriculture, College of Veterinary Medicine, Nanjing Agricultural University, Nanjing, People’s Republic of China; b Central Veterinary Institute of Wageningen UR (University & Research Centre), Lelystad, The Netherlands

## Abstract

*Streptococcus suis* is an important cause of meningitis, arthritis, and sudden death in young piglets and of meningitis in humans. A novel temperate *S. suis*-specific bacteriophage (ϕNJ2) was identified. The phage was induced from the *S. suis* strain NJ2 by using mitomycin C, and the whole genome sequence was determined. The ϕNJ2 genome is 37,282 bp in length and contains 56 open reading frames (ORFs). While 31 ORFs (55%) encoded hypothetical proteins, other ORFs were predicted to be functional, clearly indicating the novelty of ϕNJ2.

## GENOME ANNOUNCEMENT

*Streptococcus suis* infection is considered to be a major problem in the swine industry worldwide. *S. suis* can cause severe invasive infections in piglets, e.g., meningitis, arthritis, and sepsis (1). *S. suis* can also cause disease in humans. Humans are at risk mainly after contact with contaminated pigs or pig meat (2, 3).

Due to their highly specific host recognition, bacteriophages have potential as therapeutic agents in the treatment of infections (4, 5). Until now, only one lytic bacteriophage specific for *S. suis* has been described (6). In addition, several prophages could be identified in the whole genome sequences of various *S. suis* isolates (7–9). So far, however, the lytic capacities of these prophages are unknown.

Here, we report the full genome sequence of a novel temperate bacteriophage induced from the *S. suis* isolate NJ2. NJ2 was isolated from a diseased pig in China, and its temperate phage, designated ϕNJ2, was isolated from the host strain after induction with mitomycin C. The bacteriophages obtained were used for the isolation of the phage DNA. The genomic DNA of the bacteriophage ϕNJ2 was isolated using SDS-proteinase K and phenol-chloroform extraction methods (10). Genome deep-sequencing was performed using paired-end libraries (sets of two 150-bp sequences obtained with Nextera tagmentation sequencing kits [Epicentre, Madison, WI]) on an Illumina MiSeq sequencer. The quality filtered reads were subsequently assembled *de novo* using the ABySS algorithm (abyss-pe version 1.3.3), and the overall phage genome coverage was approximately 680-fold. Putative open reading frames (ORFs) were identified by GeneMarkS (11). BLASTP analyses of the putative ORFs against the NCBI nonredundant proteins (NR) database and Pfam analysis (http://pfam.sanger.ac.uk/search) were used to assess their putative functions. The prediction of tRNA sequences was carried out using the tRNAscan-SE 1.23 software (12).

The phage ϕNJ2 contains a circular double-stranded DNA genome of 37,282 bp with a *G+C* content of 39%. A total of 56 ORFs were predicted. Thirty-one of the ORFs (55%) encoded hypothetical proteins, clearly indicating the novelty of the ϕNJ2 phage. Based on Pfam homology searches, the putative functions of the putative ORFs could be categorized into five functional modules comprising proteins for (i) lysogeny (putative recombinase), (ii) DNA replication and modification (putative endodeoxyribonuclease RusA, putative C-5 cytosine-specific DNA methylase, and putative MazG nucleotide pyrophosphohydrolase), (iii) head and tail morphogenesis (phage tail protein and minor capsid protein), (iv) packaging (putative phage portal protein and putative terminase of large subunit and small subunit), and (v) host lysis (putative holin and phage amidase protein). No tRNA sequences could be identified.

The phage ϕNJ2 genome showed a high level of similarity (68% query coverage, displaying 84 to 97% identity) to sequences present in the *S. suis* isolate ST1, whereas it showed almost no similarity (less than 1% query coverage) to the lytic *S. suis* phage SMP (6).

### Nucleotide sequence accession number.

The complete genome sequence of the *S. suis* temperate bacteriophage ϕNJ2 is available in GenBank under accession number JX879087.

## References

[1] Staats JJ Feder I Okwumabua O Chengappa MM 1997 Streptococcus suis: past and present. Vet. Res. Commun. 21:381–407 9266659 10.1023/a:1005870317757

[2] Nga TV Nghia HD Tu le TP Diep TS Mai NT Chau TT Sinh DX Phu NH Nga TT Chau NV Campbell J Hoa NT Chinh NT Hien TT Farrar J Schultsz C 2011 Real-time PCR for detection of Streptococcus suis serotype 2 in cerebrospinal fluid of human patients with meningitis. Diagn. Microbiol. Infect. Dis. 70:461–467 21767702 10.1016/j.diagmicrobio.2010.12.015 PMC3146703

[3] Trottier S Higgins R Brochu G Gottschalk M 1991 A case of human endocarditis due to Streptococcus suis in North America. Rev. Infect. Dis. 13:1251–1252 1775866 10.1093/clinids/13.6.1251

[4] Harper DR Anderson J Enright MC 2011 Phage therapy: delivering on the promise. Ther. Deliv. 2:935–947 22833904 10.4155/tde.11.64

[5] Ryan EM Gorman SP Donnelly RF Gilmore BF 2011 Recent advances in bacteriophage therapy: how delivery routes, formulation, concentration and timing influence the success of phage therapy. J. Pharm. Pharmacol. 63:1253–1264 21899540 10.1111/j.2042-7158.2011.01324.x

[6] Ma YL Lu CP 2008 Isolation and identification of a bacteriophage capable of infecting Streptococcus suis type 2 strains. Vet. Microbiol. 132:340–347 18676101 10.1016/j.vetmic.2008.05.013

[7] Harel J Martinez G Nassar A Dezfulian H Labrie SJ Brousseau R Moineau S Gottschalk M 2003 Identification of an inducible bacteriophage in a virulent strain of Streptococcus suis serotype 2. Infect. Immun. 71:6104–6108 14500539 10.1128/IAI.71.10.6104-6108.2003 PMC201037

[8] Hu P Yang M Zhang A Wu J Chen B Hua Y Yu J Chen H Xiao J Jin M 2011 Complete genome sequence of Streptococcus suis serotype 3 strain ST3. J. Bacteriol. 193:3428–3429 21572001 10.1128/JB.05018-11 PMC3133292

[9] Hu P Yang M Zhang A Wu J Chen B Hua Y Yu J Xiao J Jin M 2011 Complete genome sequence of Streptococcus suis serotype 14 strain JS14. J. Bacteriol. 193:2375–2376 21398551 10.1128/JB.00083-11 PMC3133069

[10] Loeffler JM Fischetti VA 2006 Lysogeny of Streptococcus pneumoniae with MM1 phage: improved adherence and other phenotypic changes. Infect. Immun. 74:4486–4495 16861634 10.1128/IAI.00020-06 PMC1539626

[11] Besemer J Lomsadze A Borodovsky M 2001 GeneMarkS: a self-training method for prediction of gene starts in microbial genomes. Implications for finding sequence motifs in regulatory regions. Nucleic Acids Res. 29(12):2607–2618 11410670 10.1093/nar/29.12.2607 PMC55746

[12] Schattner P Brooks AN Lowe TM 2005 The tRNAscan-SE, snoscan and snoGPS web servers for the detection of tRNAs and snoRNAs. Nucleic Acids Res. 33:W686–W689 15980563 10.1093/nar/gki366 PMC1160127

